# *In vitro* antibacterial activity of curcumin-meropenem combination against extensively drug-resistant (XDR) bacteria isolated from burn wound infections

**Published:** 2020

**Authors:** Javad Yasbolaghi Sharahi, Zahra Aliakbar Ahovan, Donya Taghizadeh Maleki, Zahra Riahi Rad, Zohreh Riahi Rad, Mehdi Goudarzi, Aref Shariati, Narjess Bostanghadiri, Elham Abbasi, Ali Hashemi

**Affiliations:** 1 *Department of Microbiology, School of Medicine, Shahid Beheshti University of Medical Sciences, Tehran, Iran*; 2 *Department of Microbiology, School of Medicine, Iran University of Medical Sciences, Tehran, Iran*

**Keywords:** Curcumin, Meropenem, Extensively drug-resistant (XDR), Antibacterial, Wound Infections

## Abstract

**Objective:**

Burn wound infection is a severe complication of thermal injury. Patients with severe burn injuries need urgent care to diminish complications after severe burns. Wound infections are commonly considered one of the most serious burn complications, particularly those that are caused by extensively drug-resistant (XDR) bacteria with few therapeutic choices. The objective of this study was to determine *in vitro* activity of meropenem and curcumin, alone and in combination, against antibiotic-susceptible Gram-positive, and antibiotic-resistant and antibiotic susceptible gram-negative bacteria isolated from burn wound infections.

**Materials and Methods:**

The antimicrobial activity of meropenem and curcumin was investigated alone and in combination, against antibiotic-susceptible and antibiotic-resistant bacterial (XDR) strains isolated from burn patients. In addition, the cytotoxic effect of curcumin on human’s epithelial cell lines, was determined.

**Results:**

In this study, minimum inhibitory concentrations of meropenem decreased considerably in the presence of curcumin (2- to 16-fold reductions), with synergy observed. Curcumin exerted no cytotoxic effect at concentrations 256-512 µg/ml on human epithelial cell lines.

**Conclusion:**

We suggest that curcumin-antibiotic combinations may provide an alternative approach for treating infections with *multidrug-resistant (*MDR) and extensively drug-resistant (XDR) bacteria.

## Introduction

 Among the most prevalent and hazardous forms of trauma, burns can be singled out as a gravely traumatic experience. Patients with serious thermal injuries need urgent specialized care to reduce morbidity and mortality risks.  In severely burnt patients, more than 75% of all deaths correspond to sepsis resulting from burn-wound infections or other complications (Church et al., 2006 ▶). These infections are usually caused by bacteria such as *Streptococcus pyogenes*, *Staphylococcus aureus*, *Acinetobacter baumannii*, *Pseudomonas aeruginosa*, vancomycin-resistant *Enterococci*, and *Escherichia coli* (Betts et al., 2016 ▶). The broad spectrum of antimicrobial resistance in multi-drug resistant (MDR) and extensively drug-resistant (XDR) isolates has significantly limited effective therapeutic options (Hirsch & Tam, 2010 ▶), resulting in longer hospital stays, and higher economic burden and morbidity and mortality rates (Chew et al., 2017 ▶). Carbapenems and colistin remain part of the last-resort antibiotics for dealing with MDR Gram-negative bacteria (Chew et al., 2017 ▶). Carbapenem-resistant Gram-negative bacteria are of growing concern and have rapidly spread worldwide (McLaughlin et al., 2013 ▶). Carbapenemases mediate one of the mechanisms that confer resistance to carbapenems and have become an important cause of antibiotic resistance. A large number of carbapenemases has been detected and classified into Ambler class A (GES, KPC, NMC, IMI, and SME), class B (IMP, GIM, VIM, NDM, and SPM SIM), and class D (OXA-48) (Chiu et al., 2018 ▶). Thus, the worldwide increasing number of carbapenemase-producing bacteria has resulted in the growing use of colistin with the unavoidable risk of emerging resistance (Liu et al., 2016 ▶). Recently, Yi-Yun Liu et al. reported the emergence of plasmid-mediated colistin resistance involving *mcr-1* gene from *Klebsiella pneumoniae* and *Escherichia coli* isolates from food, animals, and humans in China (Suzuki et al., 2016 ▶). Therefore, antibacterial resistance is a serious challenge in treating burn wound bacterial pathogens, and new approaches should be applied to reduce the deaths associated with bacterial infections in burn injuries. Curcumin was first shown to have antibacterial activity in the late 1940’s. It was shown afterwards that this polyphenol possesses hypoglycemic, anti-inflammatory, wound-healing, and antioxidant activities. Extensive preclinical studies over the last decades have indicated the therapeutic capabilities of curcumin against many human diseases (Gupta et al., 2013 ▶). A combination of curcumin and other antimicrobials has proven effective in combating MDR bacteria, e.g. *A. baumannii* (Betts et al., 2016 ▶). This study investigated the *in-vitro* activity of meropenem and curcumin, alone and in combination, against antibiotic-susceptible Gram-positive (*Enterococcus faecalis*) and antibiotic-resistant and susceptible Gram-negative (*A. baumannii*, *E. coli*, *P. aeruginosa*, and *K. pneumoniae*) isolated from burn wound infections.

## Materials and Methods

**Bacterial strains and media**

Six *P. aeruginosa* positive for (*bla*_GES_, *bla*_PER-1_, *bla*_VEB_, *bla*_IMP_, and *bla*_VIM_ genes), four *A. baumannii* positive for (*OXA-51*, *OXA-24*, *OXA-23*, and *OXA-58* genes) and four *K. pneumoniae* positive for (*DHA*, *CTX-M*, *NDM-1*, and *NDM-6* genes) were isolated from wound exudate of burn patients admitted to the Burn Unit of Shahid Motahari Hospital in Tehran, Iran (2015-2017). All clinical isolates were XDR. Four standard strains, including *K. pneumoniae* ATCC 700603, *A. baumannii* ATCC19606, *P. aeruginosa* PAO1, *E. coli* ATCC25922, and *E.* *faecalis *ATCC29212, were also included in the study. The bacterial isolates were identified using conventional biochemical tests. All the bacteria isolated from burn patients, were subjected to antimicrobial susceptibility testing against commonly used antibiotics using disc diffusion method at minimum inhibitory concentration (MIC), according to the Clinical and Laboratory Standards Institute (CLSI) guidelines (Wayne, 2011). *E. coli *ATCC 25922 and *P. aeruginosa* ATCC 27853 were used as control strains in antimicrobial susceptibility testing. Curcumin (purity>65%) was obtained from Sigma-Aldrich (St. Louis, MO, USA), and antibacterial disks were purchased from Mast (Diagnostics- UK). Polymyxin E and meropenem powders from Sigma-Aldrich (St. Louis, MO).

**Detection of β-lactamase genes by PCR and Sequencing**

OXA-type carbapenemases are a major player in carbapenem resistance in clinical isolates of *A. baumannii *and they are encoded mostly by *bla*OXA-23-type, *bla*OXA-24-type, *bla*OXA-51-type, and *bla *OXA-58-type. To determine the presence of the OXA-type carbapenemase-encoding genes in the clinical isolates, a multiplex PCR method was performed, as described. (Mohammadi et al., 2017 ▶). Also, PCR and sequencing methods were used to screen the presence of *bla*_IMP_, *bla*_VIM_, *bla*_SPM_, *bla*_KPC_, *bla*_GIM_, *bla*_DIM_, *bla*_BIC_, *bla*_OXA-48, _*bla*_GES_, *bla*_VEB_, *bla*_DHA_, *bla*_CTX-15_ and *bla*_NDM_ genes in the isolates. PCR protocol used in this study was previously described (Mohammadi et al., 2017 ▶, Fallah et al., 2014 ▶). Amplification was performed in a thermal cycler (Eppendorf, Master cycler gradient). PCR product bands were analyzed after electrophoresis on 1.5% agarose gel at 100 V for 35 min in 1X *Tris/Borate/EDTA *(TBE) containing ethidium bromide under UV irradiation.

**Antimicrobial susceptibility testing**

MIC values for meropenem and curcumin were determined alone and in combination versus fourteen clinical isolates associated with burn wound infections and standard strains of Gram-negative and Gram-positive bacteria. Curcumin was diluted with 2% Dimethyl sulfoxide (*DMSO*) to yield concentrations ranging from 2 to 512 µg/ml. Checkerboard assay was carried out on 96-well microtiter plate, inoculated with MHB broth containing 5×10^5^ colony-forming units/ml of each isolate. Following 24 hr of incubation at 37°C, checkerboard assays results were recorded. The indices of fractional inhibitory concentration were assessed on the basis of the previously described method, according to which, FICa = MIC of compound a+ compound b/MIC of compound a, FICb = MIC of compound b + compound a/MIC of compound b, and FICs = FICa+FICb. FICIs≤0.5 were regarded as synergistic, values>0.5−4.0 as additive, and those >0.4 as antagonistic effects (Betts et al., 2016 ▶). All experiments were conducted in triplicates. The results are presented as mean.

**Time-kill assays**

This study employs time-kill assays to investigate and determine the antibacterial activity of mono- and combination therapies against *bla*_IMP_ harboring* P. aeruginosa* and *bla*_NDM-1 _harboring *K. pneumoniae* over a period of 24 hr. A 1/1000 dilution of an overnight culture (16 hr in Mueller-Hinton broth) (approximately 10^6^ CFU/ml) was utilized as the starting inoculum (10 ml in universal tubes) prior to adding curcumin (×1 MIC), meropenem (×1 MIC), or the combination (×1 MIC curcumin: ×1 MIC meropenem). Cultures were incubated at 37°C for 24 hr under continuous agitation (Betts et al., 2016). At the intervals of 0, 2, 4, 6, and 24 hr post-inoculation, 100 μl of the samples was collected, serially diluted and plated onto MHA. Inoculated plates were then incubated at 37°C for 24 hr and the emergent colonies were counted. Excel software was employed to plot time-kill curves (CFU/ml vs time). Of note, synergy is defined as the bactericidal activity (≥2 log10 difference in CFU/mL) of the combination as compared to the single agent after 24 hr of incubation (Betts et al., 2016 ▶).

**Cytotoxicity assay**

To investigate the percentage of cell survival following exposure to curcumin, cytotoxicity assay was carried out on human epithelial cell lines. To perform the cytotoxicity assay, human alveolar epithelial cells and fibroblast cells were cultured in RPMI 1640 medium (Biosera, USA) to which 1% L-glutamic acid, 10% fetal bovine serum (FBS), 1% non-essential amino acid and 1% penicillin-streptomycin were added. Then, cells were incubated at 37°C in a CO_2_-containing humidified atmosphere. For morphological and viability studies, at the point when the cells achieved 80% of conversion, they were seeded in 100 μl of complete medium into 96-well plates with 50,000 cells per well and, then incubated for 24 hr at 37°C with 5% CO2 to allow for the attachment of cells to the surface. Subsequently, the wells were supplemented with selected concentrations of curcumin; following 24 hr of incubation, an Olympus IX70 inverted microscope was used to evaluate cells morphology. Moreover, the microtiter wells were supplemented with various concentrations of curcumin 136 to gauge mitochondrial functions of the cells after 24 hr of seeding. Then, the wells were directly supplemented with 20 μl of XTT [2,3-bis(2-methoxy-4-nitro-5-sulfophenyl)-2H-tetrazolium-5-car-boxanilide]; after 4 hr of incubation in darkness, the absorbance at 570 nm was measure using a standard microplate reader (Anthos Labtec instruments) (Braydich-Stolle et al., 2005 ▶). 

**Statistical Analysis**

Chi-squared test was performed using SPSS 21 Software to check for any significant differences between datasets.

## Results

**Antibiotic susceptibility of clinical isolates**

Fourteen clinical isolates collected from burn patients and four standard strains were used in this study. Antibiotic susceptibility tests showed that *P. aeruginosa* and *A. baumannii* isolates were resistant to all of the currently used  antibiotics, including β-lactams (carbapenems and cephalosporins) aminoglycosides (gentamicin and amikacin), and fluoroquinolones (ciproﬂoxacin), yet remained susceptible to colistin. Three *K. pneumoniae* clinical isolates were found highly resistant to the frequently used antibiotics, i.e. carbapenems (imipenem, meropenem, ertapenem, and doripenem), extended-spectrum cephalosporins (cefoxitin, ceftazidime, cefpodoxime, and cefotaxime), aminoglycoside (gentamicin and amikacin), ﬂuoroquinolone (ciproﬂoxacin), and cotrimoxazole; three isolates were susceptible to ﬂuoroquinolone (levofloxacin), tetracycline (minocycline and tigecycline), fosfomycin, and colistin.

**Detection of carbapenemase-encoding genes**

All the isolates were resistant to all β-lactam antibiotics including carbapenems. *P. aeruginosa* clinical isolates were extended-spectrum β-lactamases (ESBL) and metallo-β-lactamases (MBL) producing. Two *P. aeruginosa *isolates were IMP-producer and one isolate was VIM-producer. In addition, two isolates of *P. aeruginosa *were GES and VEB carbapenemases-producing. The global spread of *bla*_NDM-1 _genes among multidrug-resistant bacterial pathogens is a threat to human beings that affects all patients throughout the world (Bahramian et al., 2019 ▶). In this study, three isolates of *K. pneumoniae* were NDM-producer. 

**Antibacterial activity of curcumin on clinical isolates**

MIC levels ranged from 128 to 512 μg/ml for all clinical isolates and standard strains (Table 1). These results suggest that curcumin exhibits inhibitory effect on the growth of bacteria.

**Effect of curcumin on antibiotic susceptibility**

The effect of curcumin on antibiotic susceptibility was evaluated by measuring MIC levels of meropenem with and without a sub-inhibitory concentration of curcumin. Results showed that the MICs of meropenem were significantly reduced in the presence of curcumin (2- to 16-fold reductions), with synergy observed (p ≤ 0.05) (Table 1). 

**Table 1:** Minimum inhibitory concentrations (MICs) of meropenemn and curcumin alone or in combination and fractional inhibitory concentration indices (FICIs) for potentially important pathogens of burn wounds

**Isolate**	**Genes**	**Curcumin**	**meropenem**	**Curcumin-meropenem**	FICI
		**MIC (µg/ml)**			
*K. pneumoniae*	DHA	128	128	32	0.5 (ad)
*P. aeruginosa*	VEB	128	128	32	0.5 (ad)
*A. baumannii*	OXA-23,OXA-24	128	64	16	0.37 (S)
*A. baumannii*	OXA-23,OXA-24	128	128	64	1 (ad)
*P. aeruginosa*	IMP-1	128	128	64	1 (ad)
*E. faecalis* ATCC 29212	Type strain	128	8	2	0.26 (S)
*P. aeruginosa*	GES	128	64	32	0.75 (ad)
*A. baumannii*	OXA-23,OXA-24	512	16	4	0.25 (S)
*A. baumannii* ATCC19606	Type strain	512	1	0.5	0.5 (ad)
*A. baumannii*	OXA-23,OXA-24	512	16	4	0.25 (S)
*P. aeruginosa*	IMP-1	512	128	8	0.064 (S)
*P. aeruginosa*	VIM-1	512	128	8	0.064 (S)
*E. coli *ATCC 25922	Type strain	256	0.025	0.01	0.4 (S)
*K. pneumoniae* ATCC 700603	Type strain	256	1	0.5	0.5 (ad)
*K. pneumoniae*	NDM-6	256	32	8	0.28 (S)
*K. pneumoniae*	NDM-1	256	32	16	0.56 (ad)
*K. pneumoniae*	NDM-6	256	32	16	0.56 (ad)
*P. aeruginosa*	IMP-2	256	32	16	0.56 (ad)

S = synergy, Ad = additive effect.

**Synergism of curcumin with antibiotic susceptibility**

**Figure 1:**
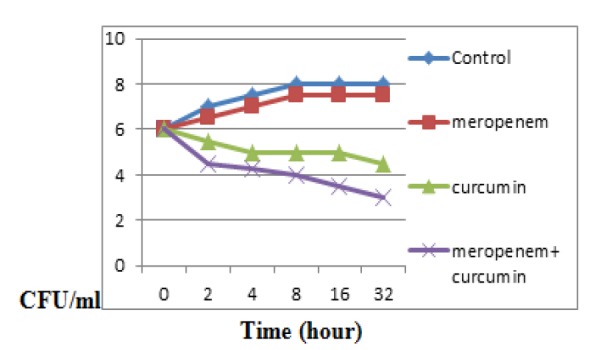
Time−kill curves plotted for meropenem and curcumin alone and in combination against *K.* *pneumoniae* (NDM)

In order to explore the interaction between curcumin and meropenem, checkerboard assay was accomplished to define the FIC indices for both carbapenem-associated multidrug-resistant isolates and also the type strain. FIC indices synergy between curcumin and meropenem ranged from 0.064 to 1 (Table 1). Additionally, to confirm the synergism of curcumin with the meropenem, time-killing assays were performed for *K. pneumoniae* positive for (*bla*_NDM-1 _gene). As shown in Figure 1, NDM-producing *K. pneumoniae* grew within 24 hr with inhibitory concentrations of meropenem alone and in combination with curcumin. However, over 99.99% of NDM-producing *K. pneumoniae* were killed within 24 hr with the same concentrations of both the meropenem and curcumin. These results imply that the interaction of curcumin with meropenem is synergism (*p* ≤ *0.05*).

**Cytotoxicity assay**

Morphology of the cell lines was investigated after 24 hr of incubation with different concentrations of curcumin (256-512 µg/ml). The results showed that the human alveolar epithelial cells were well spread, and there was no distinct change in cell morphology after 24 hr of incubation with various concentrations of curcumin relative to control cells.

## Discussion

Broad spectrum antimicrobial resistance in MDR and XDR isolates significantly limited effective therapeutic options (Hirsch & Tam, 2010 ▶). The present study evaluated an alternative approach to treat the antibiotic-susceptible and -resistant Gram-positive (*E. faecalis*) and Gram-negative (*A. baumannii*, *E. coli*, *P. aeruginosa*, and *K. pneumoniae*) isolates associated with burn wound infections. As expected, the prevalence of carbapenem-associated multidrug-resistance among the clinical isolates in Iran was high. In this study, all of the isolates were resistant to carbapenems and the only effective antibiotic was colistin. Polymyxins are the last-resort options to treat carbapenem-associated multidrug-resistant isolates. Since the beginning of the 21st century, an increasing number of carbapenem-resistant clinical isolates has been reported around the world along with increasing reports on polymyxin-resistant isolates (Liu et al., 2016 ▶, Lee et al., 2017 ▶). Carbapenem- and polymyxin-associated multidrug-resistant isolates of *P. aeruginosa*, *K. pneumoniae* and *A. baumannii *are now common in Iran. Although a number of therapeutic options have been suggested to combat the carbapenem- and polymyxin-associated multidrug-resistant isolates (Menegucci et al., 2016 ▶) , none of them has proven fully satisfactory for treating these extensively drug-resistant or pan-drug-resistant isolates. *Curcuma longa* has been traditionally used in Asian countries as a medical herb due to its anti-inflammatory, antioxidant, antimicrobial, antimutagenic, and anticancer properties (Hewlings & Kalman, 2017 ▶). Curcumin has also been shown to have *in vitro* anti-microbial effects against a wide range of microorganisms including fungi as well as several Gram-negative and Gram-positive bacteria (Tyagi et al., 2015 ▶). Negi et al. reported that curcumins and curcuminoids possess better antibacterial activity against a wide range of microbes including *Staphylococcus aureus, Bacillus cereus, Bacillus subtilis, Bacillus coagulans, P. aeruginosa* and *E. coli *(Gul & Bakht, 2015 ▶). Karaman et al. showed that treatment of strains with MIC and sub-MIC concentrations of curcumin did not significantly increase the optical density of biofilm. The data obtained in our study supported the promising inhibitory effect of curcumin on *P.aeruginosa* biofilms (Karaman et al., 2013 ▶). In this study, minimum inhibitory concentrations of meropenem were significantly (p≤0.05) reduced in the presence of curcumin (2- to 32-fold reductions), with synergy observed. Combination therapy with different antibiotics has been used to combat *A*. *baumannii* infections. Kaur et al. showed that curcumin significantly decreased persistence against colistin. Hence, curcumin-colistin combination can be another option with anti-persister potential to control chronic *A*. *baumannii* infections (Kaur et al., 2018 ▶). Betts et al. reported the antimicrobial synergy between polymyxin E and curcumin and suggested that a combination of the two compounds could be used to treat or prevent traumatic wound infections of the skin (Betts et al., 2016 ▶). Another compound that has been found effective on *A. baumannii *in combination with meropenem, is epigallocatechin-3-gallate (EGCG). We confirmed the antibacterial activity of curcumin on carbapenem-associated multidrug-resistant clinical isolates, as shown by the MIC (256-512 μg/ml) of curcumin. These results are consistent with the notion that curcumin alone shows antibacterial activity on clinical isolates of *A. baumannii*, regardless of the presence of antibiotic resistance genes (Lee et al., 2017 ▶). In another study, Bansal et al. reported that curcumin alone and in combination with augmentin, could protect against pulmonary inflammation and acute lung injury generated during *K. pneumoniae* B5055-induced lung infection in BALB/c mice (Bansal & Chhibber, 2010 ▶). It was also found that curcumin synergistically increased *A. baumanii *susceptibility to meropenem in all tested carbapenem-associated multidrug-resistant isolates. Synergistic effect of curcumin on antibiotics in *A. baumannii *(Kaur et al., 2018 ▶) has been reported. The obtained results demonstrated that antibacterial activity of curcumin and its synergism with meropenem can sensitize carbapenem-associated multidrug-resistant isolates of *A. baumannii*, *P. aeruginosa*, and *K. pneumoniae*. Overall, we suggest that curcumin-antibiotic combinations may provide an alternative approach to treat infections with MDR and XDR bacteria regardless of antibiotic resistance.
